# Reduction in the duration of postoperative fever following NUSS surgery during the COVID-19 pandemic

**DOI:** 10.1186/s13052-023-01524-6

**Published:** 2023-09-13

**Authors:** An Jia, Wang Qiang, Deqin Zhuoga, Yu Di, Yang Zhaocong, Mo Xuming

**Affiliations:** 1 Nanjing Children’s Hospital, Clinical Teaching Hospital of Medical School, Nanjing University, Nanjing, China; 2 Children’s Hospital of Nanjing Medical University, Nanjing, China

**Keywords:** Postoperative fever, NUSS procedure, pectus excavatum, COVID-19, Surgical mask

## Abstract

**Background:**

Our study aimed to compare the prevalence of postoperative fever during the COVID-19 pandemic period with that of the preceding non-pandemic period.

**Methods:**

A retrospective analysis was conducted on patients with pectus excavatum (PE) undergoing minimally invasive repair (also called NUSS procedure) at Nanjing Children’s Hospital from January 1, 2017 to March 1, 2019 (Group 2019), and from January 1, 2020 to March 1, 2021 (Group 2021). Data from a total of 284 patients, consisting of 200 (70.4%) males and 84 (29.6%) females with an average age of 9.73 ± 3.41 (range, 4 to 17) years, were collected. The presence of post-operative fever (defined as a forehead temperature of 37.5℃ or above within 72 h post-surgery), as well as the time of operation, duration of postoperative mechanical ventilator and urinary catheter use, and length of hospitalization were all assessed in admitted patients from Group 2019 (*n* = 144) and Group 2021 (*n* = 140). Postoperative white blood cell (WBC), C-reactive protein (CRP) levels, and prevalence of postoperative complications (i.e., pneumothorax, pulmonary atelectasis, pneumonia, wound infection, and dehiscence) were also determined.

**Result:**

Our results showed a statistically significant decrease in the incidence of postoperative fever within 24 to 72 h of surgery in patients admitted from Group 2019 as compared to Group 2021 (*p* < 0.001), as well as a decrease in peak body temperature within 72 h (*p* < 0.05). Meanwhile, no significant differences were observed in age and body mass index (BMI), time of operation, or duration of postoperative mechanical ventilator and urinary catheter use between the two groups (*p* > 0.05). The average hospitalization length of Group 2021 was significantly shorter than Group 2019 (12.49 ± 2.57 vs. 11.85 ± 2.19 days, *p* < 0.05). Furthermore, while the WBC count between the two groups 24 h after surgery showed a statistical difference (*p* < 0.05), no differences in CRP levels or the incidence of postoperative complications were observed (*p* > 0.05).

**Conclusion:**

The prevalence of postoperative fever within 72 h of surgery and the length of hospital stay for patients with PE undergoing NUSS surgery were both decreased in Group 2021. We propose that the above phenomenon may be related to increased used of personal protection equipment (such as surgical masks and filtering facepiece respirators (FFRs)) by physicians, nurses, and the patients themselves.

**Supplementary Information:**

The online version contains supplementary material available at 10.1186/s13052-023-01524-6.

## Introduction

Pectus excavatum (PE) is the most common congenital birth defect of the chest wall that often causes psychological and social issues [1], limited exercise capacity [2], and even decreased sleep quality. PE occurs in 1 out of every 400–1000 births. The first reports of corrective surgery to fix PE date back a century, as Meyer and colleagues first reported the surgical treatment of PE in 1911 [3]. Currently, the most accepted means of correcting PE is through minimally invasive surgical repair (NUSS procedure) [4]. Importantly, postoperative fever is a common symptom in the early postoperative period following NUSS. Fever onset may be caused by metal allergies [5], hematoma absorption, transfusion reactions, or result from unknown origins. Clinically, fever is one of the common postoperative symptoms and does not seem to be significantly associated with serious complications. As such, fever has not received much attention. When the first case of pneumonia of unknown cause was reported on 8 December 2019 in Wuhan, China [6], within one month, the Chinese Center for Disease Control and Prevention had successfully isolated and named the novel strain of coronavirus, and by 7 January 2020, the National Health Commission (NHC) had issued guidelines for epidemic prevention and control. To prevent COVID-19 in public, several prevention tools, such as use of surgical masks and filtering facepiece respirators (FFRs), were introduced into daily clinical practice [7]. Meanwhile, for high-risk occupational exposure groups, such as hospital workers, the NHC recommended that doctors and nurses should wear overalls, work hats, and medical-grade surgical masks. In addition, relatives of patients were limited or forbidden to visit inpatient departments. During this time period, our cardio-thoracic surgery department observed an empirical decrease in the incidence of post-operative fever in patients admitted for PE.

As the duration of the surgical procedure, protocols for prophylactic administration of antibiotics, and other possible causes related to postoperative fever were all the same, we hypothesized that the lower incidence of postoperative fever in Group 2021 was related to the increased precautions used during hospitalization in the early period of COVID-19, specifically, the use of personal protective equipment (PPE) and no external visits to the hospital.

The aim of this study was to compare the incidence of postoperative fever during the COVID-19 pandemic (January 1, 2020 to March 1, 2021) (Group 2021) to that of the preceding non-pandemic period (January 1, 2017 to March 1, 2019) (Group 2019).

## Materials and methods

### Patient cohorts

A single-center retrospective observational cohort study was conducted on all of the NUSS procedures performed for PE patients at Nanjing Children’s Hospital, China from January 1, 2017 to March 1, 2019 (Group 2019), and from January 1, 2020 to March 1, 2021 (Group 2021). We analyzed 142 admitted patients in Group 2021. While all admitted patients were hospitalized for NUSS surgery, 12 had scoliosis, 9 exhibited a flat chest, and 2 presented with fever prior to surgery were excluded. In the non-pandemic period (Group 2019), 144 patients were screened, and 8 patients with scoliosis and 6 patients with a flat chest. Clinical data, including demographic information, surgical details, and discharge summaries were gathered retrospectively based on available medical records. Baseline characteristics were collected, including age, gender, body mass index (BMI, kg/m^2^), as well as the duration of surgery, mechanical ventilator, and urinary catheter use. Forehead temperature was measured within 72 h after surgery, and a postoperative assessment of white blood cell (WBC) count, C-reactive protein (CRP) levels, and incidence of postoperative complications (such as pneumothorax, pulmonary atelectasis, pneumonia, wound infection, and dehiscence), as well as length of the hospital stay were measured and recorded.

### Postoperative fever (definition and workup)

Body temperature was assessed post-surgery every 4 h via checking forehead temperatures. Fever was defined as a forehead temperature of 37.5℃ or above within 72 h following surgery.

### Statistical analysis

Patient characteristics are presented as mean ± standard deviation or median (interquartile range, P_25_-P_75_) for continuous variables with normal distribution; n and categorical variables were presented as a percentage (%). Continuous variables were examined using Student’s t test and Wilcox signed-rank test. Categorical variables were compared using the Pearson chi-squared test. Data were analyzed using the Statistical Package for Social Sciences software (version 26.0, SPSS Inc., Chicago, USA). A *p* < 0.05 was considered statistically significant.

## Results

**Fig. 1:**
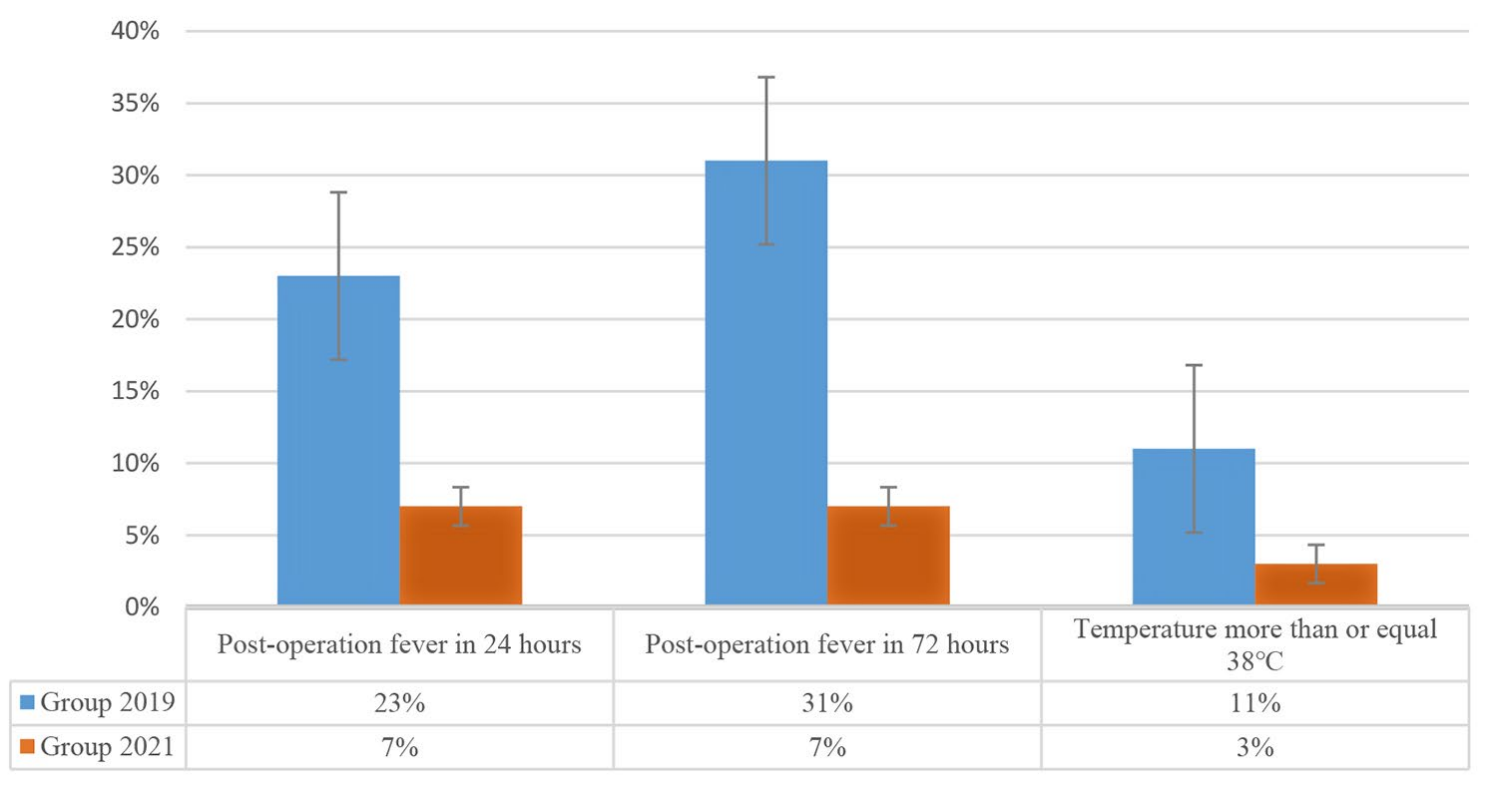
Posteroperation fever between two groups

Data from a total of 284 patients, consisting of 200 males and 84 females with an average age of 9.73 ± 3.41 (range, 4 to 17) years, were collected. 144 patients were allocated into Group 2019, with an average age of 10 years (range 6 to 13) and a mean BMI of 15.68 ± 2.46 kg/m^2^. Alternatively, 140 patients were allocated into Group 2021, with an average age of 11 years (range 8 to 13) and a mean BMI of 16.25 ± 12.92 kg/m^2^. Age and BMI were similar between the two groups (*p* > 0.05) (Table 1). The duration of the surgical procedure, as well as mechanical ventilator and urinary catheter use were also no difference between the two groups (*p* > 0.05) (Table 1). Contrastingly, the prevalence of postoperative fever 72 h after surgery in Group 2021 was significantly lower than Group 2019 (7% vs. 31%, *p* < 0.001)(Fig. 1). In Group 2021, fever was observed in the first 24 h post-surgery in 11 (7%) patients, which was also the percentage observed 72 h after surgery. In Group 2019, fever was observed in the first 24 h post-surgery in 35 (23%) patients, which increased to 47 (31%) patients after 72 h. In addition, the peak postoperative fever temperature recorderd at 72 h was also significantly higher in Group 2019 (*p* < 0.05) (Table 1). Accordingly, the length of hospitalization in Group 2021 was also significantly shorter than that of Group 2019 (*p* < 0.05) (Table 1). We also observed a statistically significant difference in the level of WBC between the two groups at 24 h after surgery (*p* < 0.05) (Table 1). However, no statistical difference were observed in CRP level or the incidence of postoperative complications between the two groups (*p* > 0.05) (Table 1).

**Table 1:** Clinical characteristics of PE patients and comparison between the two groups

Variables	Group 2019 (*n* = 144)	Group 2021 (*n* = 140)	*P*
Gender, female	44(30%)	40(28%)	0.82
Age (years)	10(6, 13)	11(8, 13)	0.11
BMI(kg/m^2^)	15.68 ± 2.46	16.25 ± 12.92	0.61
Duration of the surgical procedure (minutes)	55.57 ± 25.57	55.30 ± 18.77	0.91
Duration of the mechanical ventilator (minutes)	71.67 ± 48.23	63.69 ± 27.03	0.08
Duration of the urinary catheter use (hours)	20.65 ± 4.22	21.37 ± 6.91	0.28
Post-operation fever in 24 h	35(23%)	11(7%)	< 0.001*
Post-operation fever in 72 h	47(31%)	11(7%)	< 0.001*
Temperature more than or equal 38.0℃	17(11%)	5(3%)	0.01*
WBC, ug/ml	12.69 ± 4.03	11.76 ± 2.53	0.02*
CRP, ug/ml	14.76 ± 13.59	12.07 ± 9.01	0.13
Prevalence of complications	18(12%)	20(14%)	0.595
Length of hospitalization (days)	12.49 ± 2.57	11.85 ± 2.19	0.02*

Statistically significant data in bold (*p* < 0.05). BMI [16], Body Mass Index = weight/height^2^, BMI ≥ 18.5 to 24.9 kg/m^2^ means normal weight, BMI < 18.5 kg/m^2^ means underweight, BMI < 16.5 kg/m^2^ means severely underweight, BMI ≥ 25 to 29.9 kg/m^2^ means overweight, BMI ≥ 30 kg/m^2^ means obesity

## Discussion

Our study shows a significant decrease in the post-operative fever of patients in Group 2021 compared to Group 2019 (7% vs. 31%, *p* < 0.001). Furthermore, we observed a reduction in the postoperative fever temperature (degree of fever ≥ 38.0℃) in Group 2021 (3% in Group 2021 vs. 11% in Group 2019, *p* < 0.05). Meanwhile, we also observed a statically significant difference in postoperative WBC count between the two groups, while CRP levels remained unchanged. Interestingly, we observed that infection-related complications did not decrease with the declining fever rate, possibly because the complication rate after surgery was very low. During the entire observation period, only 1 in 284 patients was found to have poor wound healing (a small amount of pink exudation), and the wound became better after daily dressing changes and systemic prophylactic antibiotic therapy. Data in the literature indicate that the incidence of wound healing after NUSS surgery is 1.5–6.9% [8]. During the COVID-19 epidemic, where the prevalence of postoperartive fever decreased significantly within 72 h post-surgery, we were unable to observe any decrease in the incidence of complications, although the hospital stay was significantly shortened, resulting in lower healthcare costs.

Fever is a common complication in the early stages following the NUSS procedure, and fever is often associated with infection-related complications. We hypothesize that the decline in the frequency of postoperative fever observed during the epidemic was most likely related to the strengthening of personal protection measures for physicians, nurses, patients, and family members, including implementation of surgical masks, FFRs, and limiting the number of relatives allowed into inpatient departments, thus reducing the risk of pathogen exposure [9–11]. However, due to our failure to perform preoperative and postoperative respiratory tract or blood tract tests on patients with PE, we were unable to objectively obtain evidence of pathogen transmission. Wilder et al [12]found that during the COVID-19 epidemic, pediatric hospitalization rates for virus-related acute diseases (such as asthma, bronchitis, and pneumonia) decreased, suggesting that measures such as wearing surgical masks can reduce the spread of the virus significantly. According to statistics, the frequency of postoperative fever ranges from 18 to 60%. Since fever is a common symptom after surgical procedures, it previously did not garner much attention. However, since the COVID-19 epidemic, fever has become one of the more frequent screening factors to identify viral infections, which has significantly increased fever awareness [13], and the correlation between fever temperature and the prognosis of viral infections has been reported in a few studies. In addition, we accept that fever can reflect the presence of an inflammatory reaction in the body, however, fever may not be related to the severity of inflammation. We routinely tested serum from the two groups at the same time and significant differences were only found in the WBC count, but not CRP levels. Thus, it is difficult to conclude that fever is associated with the degree of inflammation.

Furthermore, while BMI was not significantly different between the two groups (*p* > 0.05), the BMI of both groups was lower than the normal range, with some of the patients being severely underweight. This supports the notion that PE is more likely to occur in thin children, which has been reported previously [14]. Therefore, adolescents should be encouraged to eat a balanced diet, strengthen their exercise routine, and enhance their physical fitness.

As the present study was limited to PE patients, additional surgery types have not yet been investigated, which makes it impossible to as of yet infer the broader implications of the observed postoperative fever phenomenon that arose during the COVID-19 epidemic. Interestingly, a previous clinical study carried out in another single center also found a decrease in the postoperative fever rate [15]. In this study by Mastri and colleagues, they showed that the other observation objects were not involved in the NUSS postoperative group. Thus, this study provides more convincing evidence for the phenomenon we have observed. However, further studies with larger cohorts at multiple clinical research centers is required for subsequent validation of our results.

## Conclusion

In the early stages of the COVID-19 epidemic, the absence of rapid and precise screening tests for COVID-19 significantly increased concern regarding clinical symptoms such as fever and cough. However, this troublesome situation led to stronger measures to prevent respiratory infections (surgical masks and FFRs), also reducing the spread of the virus to the public. As for PE patients, there was a significant decrease in the prevalence of postoperative fever and the length of hospitalization, further decreasing the overall economic cost.

### Electronic supplementary material

Below is the link to the electronic supplementary material.

Supplementary Material 1

## Data Availability

The data presented in this study are available on request from corresponding author.

## List of Abbreviations

PE: Pectus excavatum
WBC: White blood cell
CRP: C-reactive protein
BMI: Body mass index
FFRs: Filtering facepiece respirators
NHC: National Health Commission
PPE: Personal protective equipment
