# Supplementary material for: Reduction in the duration of postoperative fever following NUSS surgery during the COVID-19 pandemic
Source: Ital J Pediatr. 2023 Sep 13;49:120. doi: 10.1186/s13052-023-01524-6 (PMC10500851; doi:10.1186/s13052-023-01524-6)
Supplement: Supplementary file 1 — Supplementary Material 1 [file 13052_2023_1524_MOESM1_ESM.docx]

Dear editor

# We wish to submit our article “**Reduction in the duration of postoperative fever following NUSS surgery during the COVID-19 pandemic** ”to be considered for publication in **Italian Journal of Pediatrics** .

# During the COVID-19 pandemic, we found that the face protection could reduce the postoperative fever rate. We chose to conduct an observational study on patients with pectus excavatum (PE) and the conclusions showed that the fever rate of postoperative children with PE during the COVID-19 pandemic was significantly reduced. With the end of the epidemic, is it essential to continue to have face protection, at least in hospitals.We think this article will be of interest to your readers as it bring new ideas and pave the way for clinical benefit.

I am authorized on behalf of all the authors of this article to confirm that authors has any conflict of interest to disclose, all authors have approved the version submitted for publication, the work in this article is original and has not been published previously, and the article is not under consideration by any other journal.

We thank you for your king consideration of our article. Please do not hesitate to contact me directly if any further information is required. I look forward to hearing from you.

Your sincerely
